# Feasibility of on-demand robotics in colorectal surgery: first cases

**DOI:** 10.1007/s00464-023-10284-7

**Published:** 2023-07-24

**Authors:** Dieter Hahnloser, Djana Rrupa, Fabian Grass

**Affiliations:** Department of Visceral Surgery, University Hospital Lausanne, CHUV, Rue du Bugnon 46, CH-1011 Lausanne, Switzerland

**Keywords:** Robotics, Colorectal, Laparoscopy, Robotic surgery, Dexter

## Abstract

**Introduction:**

The key benefits of robotics are improved precision and control, thanks to fully articulated robotic instruments and enhanced, stable endoscope control. However, colorectal procedures also require large movements such as medialization of the colon where a robotic platform is not always needed. We present the world’s first experience in colorectal surgery with a new open platform of on-demand robotics.

**Methods and procedures:**

Standard laparoscopic 3-D camera, insufflator, trocars and energy devices, available in all hospitals performing laparoscopic surgery, are used in combination with the Dexter System™ from Distalmotion SA, which includes two robotic instrument arms, one robotic endoscope arm and a sterile surgeon console. We present the first 12 colorectal cases of robotic assisted ventral mesh rectopexy (*n* = 2), oncologic right colectomies (*n* = 8), transverse colectomy (*n* = 1) and ileocecal resection (*n* = 1) using the Dexter System.

**Results:**

The two ventral mesh rectopexies were fully robotic, requiring no switching from standard laparoscopy to robotic assistance. The robotic platform was used for central vascular ligation (CVL) in all 8 oncologic colectomies, whereas medialization of the colon and transection was performed with standard laparoscopy. The switch from laparoscopy to robotics and back was performed in 15–30 s. Intracorporal anastomosis was performed in 4 patients (stapling by standard laparoscopy and suturing of the defect with robotic assistance). Conversion or permanent switch to standard laparoscopy was required in two patients due to visceral obesity. No robotic platform-related intraoperative adverse event occurred. No major morbidity occurred at 60 days.

**Conclusions:**

On-demand robotics is feasible and combines the best of two worlds: Robotics where precision and enhanced dexterity are required and standard laparoscopy where it is at its best. The surgeon remains scrubbed-in at all times, allowing a switch between robotics and laparoscopy within seconds.

Thanks to improved dexterity, visualization and ergonomics, robotic platforms are increasingly used for complex surgical procedures. In the US, the adoption of robotic surgery is increasing and associated with a decrease in postoperative complications and length of stay [1, 2]. However, several drawbacks related to the high cost of acquisition and maintenance, logistic prerequisites such as dedicated operating rooms and teams, and availability in multidisciplinary practices impede widespread implementation outside high volume institutions [3].

In colorectal surgery, the robot has particularly proven its value when dissecting restrained spaces such as the male pelvis, or when increased precision is required during suturing or central vascular dissection [4–6]. However, for other procedure steps such as lateral to medial mobilization of the colon, omental release or splenic flexure takedown, the robot may be less mandatory and laparoscopy more suitable, since dissection is carried out in large spaces needing rapid navigation and frequent position changes. This mindset to offer both techniques during the same operation led to the concept of “on-demand” robotics, where both techniques can be interchangeably used during the same procedure [7, 8].

The present study presents the world’s first clinical experience of the Dexter On-demand robotic platform in colorectal surgery.

## Material and methods

### Dexter robotic system™

**Fig. 1:**
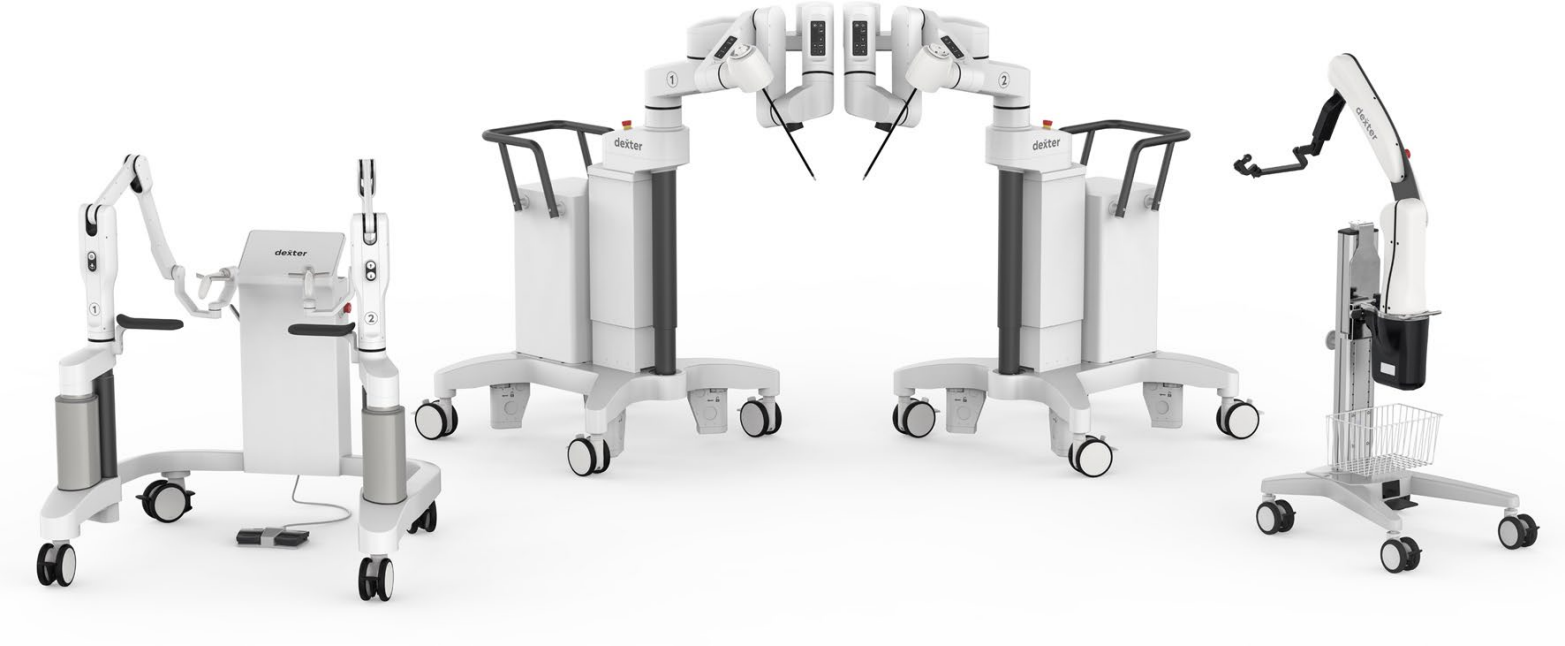
Dexter Robotic System™ with one surgeon console, two patient carts and one camera arm. Dexter is an open platform and compatible with any 3D endoscope, RF generator and insufflator

The new Dexter Robotic System™ (Distalmotion SA, Switzerland) is an open robotic platform designed to optimize how to perform minimally invasive surgery. The system consists of (1) a surgeon console that can remain sterile, (2) two patient carts actuating a range of instruments with seven degrees of freedom and 75 degrees angulation and (3) a robotic endoscope arm able to host any 3D endoscopic system and fully controllable from the surgeon console (Fig. 1).

**Fig. 2:**
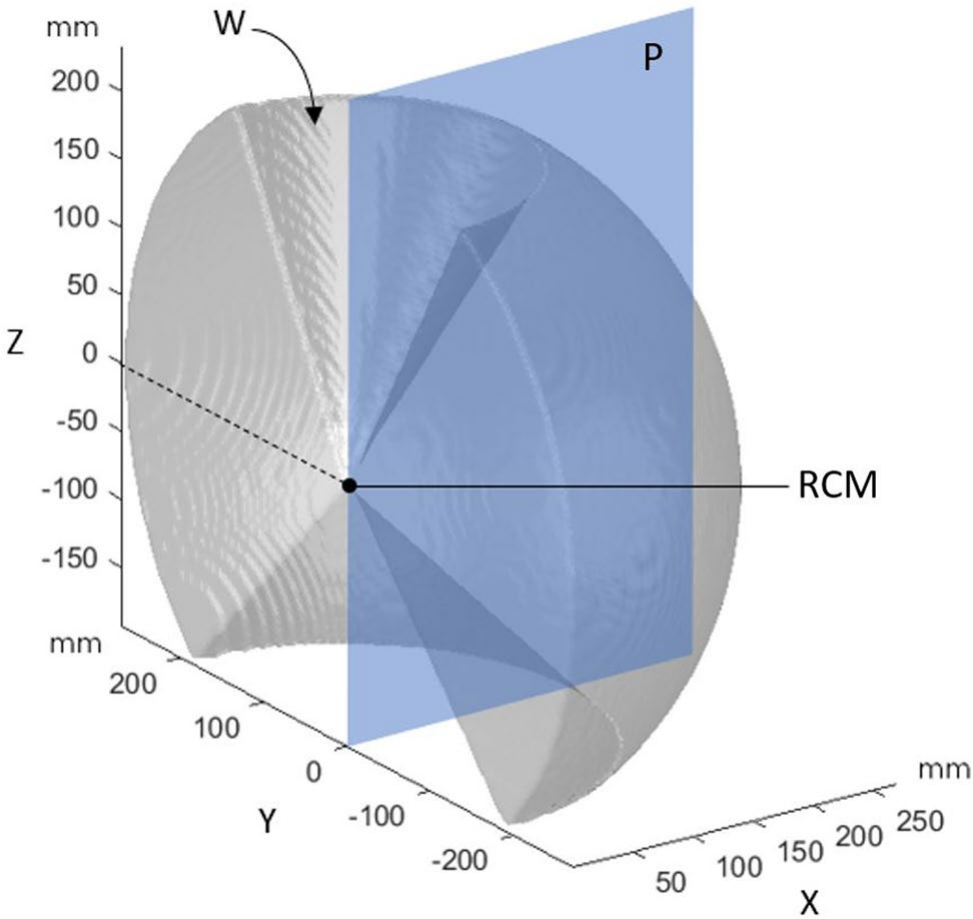
3D view of the Dexter robot’s workspace (denoted W), i.e., the area where surgery can be performed with respect to the trocar entry point (denoted RCM for remote center of motion)

**Fig. 3:**
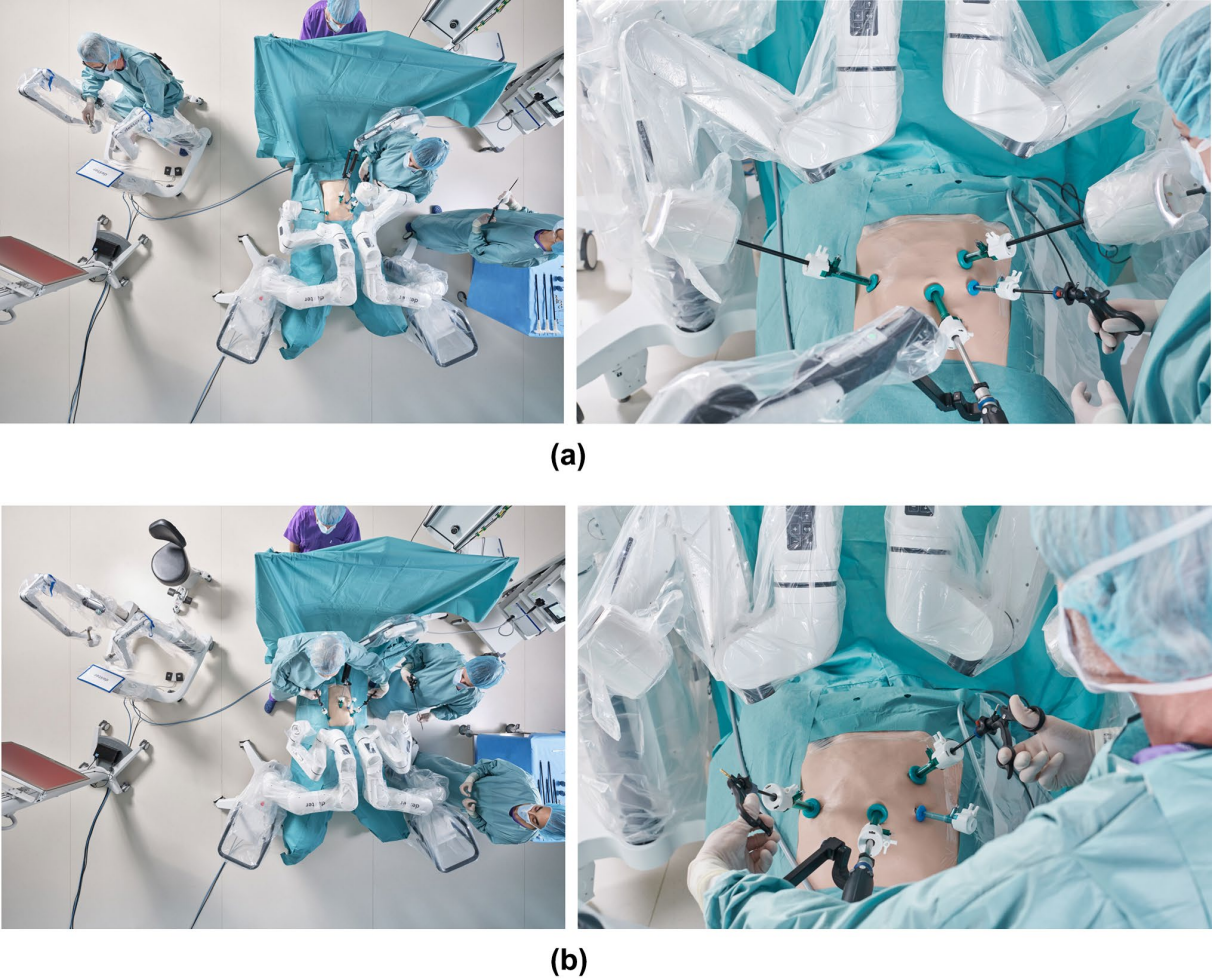
Modality switch during on-demand robotics. (**a**) Top view and front view of a typical Dexter surgical setup in robotic mode. (**b**) Top view and front view of a typical Dexter surgical setup in laparoscopic mode. Both instrument arms are folded in order to create sufficient space for standard laparoscopy

The current portfolio of instruments includes a needle holder, a Johann grasper, a Maryland bipolar dissector, monopolar scissors and a monopolar hook (8.3 mm instruments). Workspace is depicted in Fig. 2. The Dexter system is an open system and integrates into the existing operating room (OR) equipment suite. Any available RF generator, insufflation device and 3-D optics can be connected to Dexter. The trocars positioning for Dexter is similar to what is used in standard laparoscopic surgery. An assistant trocar may be positioned for the assistant to manipulate instruments such as retractors, irrigation and suction device as he would do in a standard laparoscopic surgery setup. The small profile of the system provides space for surgical assistants and nurses to access the patient during robotic operation (Fig. 3). The robotic arms are designed to facilitate docking and instrument change. For switching to laparoscopic mode, one button commands the folding of the robot arms leaving sufficient space to the surgeon to operate in traditional laparoscopic setup without undocking the robot (Fig. 3).

### Patient population and OR set-up

We describe the first consecutive colorectal patients operated with Dexter in our institution. All patients signed the general informed consent form of the hospital allowing use of all clinical data for research purposes. Dexter is CE marked and indicated for general surgery, gynecology, and urology. This is a feasibility and safety study.

Standard laparoscopic 3-D camera, insufflator, trocars and energy devices were used in combination with Dexter. Trocar set-up for right colectomy and for ventral mesh rectopexy were similar to trocar positions for standard laparoscopic procedures. One 12 mm camera trocar and three 11 mm standard trocars were used. Patients were in modified lithotomy position with the arms along the body. The two patient carts were placed on each side of the patient and the camera arm on left of the patient for rectopexy and alongside the left leg for right colectomy.

## Results

In total, 12 patients underwent eight oncologic right colectomies, two ventral mesh rectopexies, one oncologic transverse segmental colectomy and one ileo-cecal resection with extended mesenteric resection using the Dexter system (Table 1). The two procedures of ventral mesh rectopexies were fully robotic, requiring no switch from standard laparoscopy to robotic assistance.

**Table 1:** Consecutive patients operated with Dexter

	Gender/ age	Diagnosis	Intervention	Total OR time (min)	Robotic steps	Conversion	Intraoperative adverse events	Length of stay (days)	60 days complication (grade Clavien- Dindo)
1	F/77	ODS	VMR	186	Fully	No	None	3	-
2	M/81	Adenocarcinoma	R col	140	CVL	No	2 additional trocars due to bad exposure	8	Ileus (II)
3	F/67	Sarcoma colon	R col	122	CVL	No	None	6	Ileus (II)
4	M/62	Adenocarcinoma	Transverse col	135	CVL	No	None	15	Ileus (II)
5	F/73	ODS	VMR	149	Fully	No	None	6	–
6	M/64	Polyp	R col	199	CVL ICA	No	None	5	–
7	M/44	Chronic colitis	R col	108	CVL	Yes to lap. (visceral obesity)	None	7	Ileus (II)
8	M/83	Adenocarcinoma	R col	206	CVL ICA	No	None	18	Anastomotic bleeding (IIIa)
9	M/76	Adenocarcinoma	R col	142	CVL	No	None	18	Anastomotic leak (II)
10	F/79	Polyps	R col	147	CVL	No	None	7	–
11	M/64	Adenocarcinoma	R col	204	CVL ICA	Yes to lap. (visceral obesity)	None	7	SSI (I)
12	F/18	Crohn	ICR	152	CVL ICA	No	None	6	Ileus (II)

*F* female, *M* male, *ODS* Obstructed Defecation Syndrome, *VMR* ventral mesh rectopexy, *R col*. oncologic right colectomy, *CVL* Central vascular ligation, *ICA* Intra-corporeal anastomosis, *SSI* Superficial Site Infection. *ICR* ileo-cecal resection with extended mesenteric resection, *lap*: laparoscopy

**Fig. 4:**
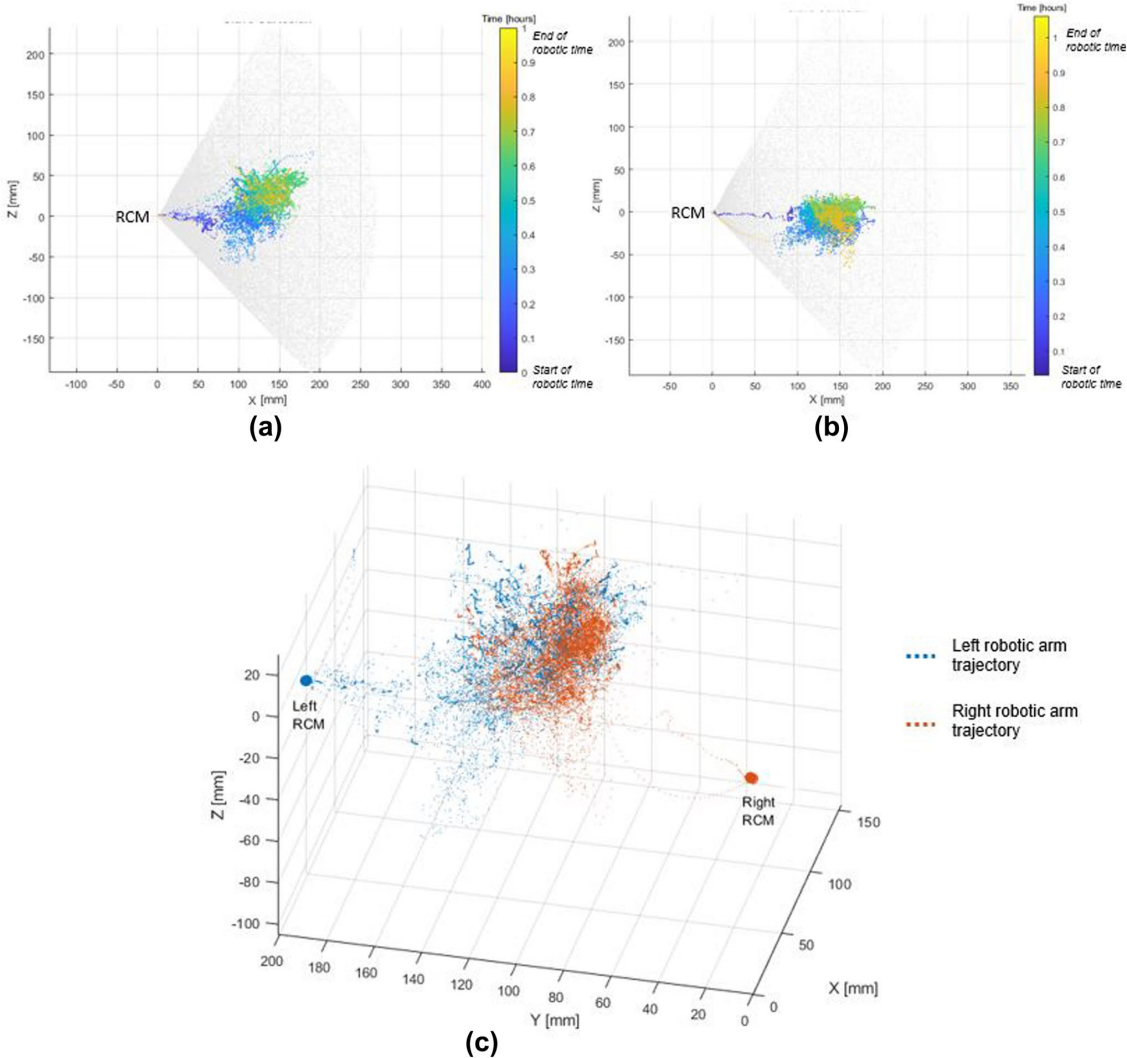
Workspace robotic arm trajectories of a right colectomy performed with the Dexter System. (**a**) 2D projection of left robotic instrument arm trajectory over time. Gray dots represent total possible workspace. RCM (remote center of motion) represents trocar entry point (**b**) 2D projection of right robotic instrument arm trajectory over time. (**c**) 3D trajectories of right and left robotic instrument arms over the course of the procedure (Color figure online)

For oncologic resection a vascular first approach was used. Central vascular dissection and ligation (CVL) was performed with Dexter harvesting between 15 and 28 lymph nodes. The ileocolic vessels were clipped with Hemolok (Weck, USA). Lateral to medial mobilization of the right colon was performed by switching to standard laparoscopy using the same trocars (Fig. 3). The switch took between 15 and 30 s. For intracorporeal anastomosis (ICA, in 4 patients) the colon and ileum were stapled using standard laparoscopic 60 mm staplers fashioning a side-to-side isoperistaltic anastomosis. After switching to Dexter the defect was closed with running 3–0 absorbable V-lock (Medtronic, USA) using the robotic instruments. Figure 4 shows an example of trajectories of both robotic instrument arms within their workspace during a right colectomy performed in our institution, illustrating ample workspace and the absence of any movement limitation during robotic manipulation. Extraction of the specimens was performed through a Pfannenstiel incision. Extracorporeal anastomoses were performed through a periumbilical incision in a side-to-side an-isoperistaltic fashion [9]. The choice for intra- or extracorporeal anastomosis was at the surgeon’s discretion.

Two conversions or permanent switch to standard laparoscopy were necessary due to visceral obesity using an additional trocar. No conversion to open surgery occurred. In one patient three additional trocars needed to be placed for better exposure. The CVL could be performed with Dexter afterwards. No Dexter-related intraoperative adverse event occurred. One anastomotic bleeding was clipped by colonoscopy and one anastomotic leakage was treated by antibiotics. All Ileus were treated by nasogastric tube insertion and bowel rest. No patient needed re-operation within 60 days. Ileus can occur in up to 24.7% [10], but were not related to the uses of Dexter, as CVL and anastomoses were performed in the same way as we do in standard laparoscopy.

## Discussion

This single center study presents first encouraging results in colorectal surgery after implementation of the Dexter on-demand robotic platform. All procedures were carried out without major morbidity. We observed no technical issues related to the robotic platform, allowing switches between standard laparoscopy and robotics within seconds.

The Dexter platform was developed in an intent to offer the surgeon the possibility to benefit from both approaches within the same procedure: on the one hand, robotics where precision and dexterity are required providing articulated instruments and full endoscopic control of the camera. On the other hand, the standard laparoscopic approach with already available standard OR equipment in any center performing minimally invasive surgery. Hence, in contrast to a full robotic platform competing with and replacing standard laparoscopy, Dexter offers a flexible on-demand approach, combining the best of both worlds. Dexter ensures procedural fluidity. Both instrument arms were designed to optimize movement stability and to maximize the available workspace, in order to remove movement limitation for procedures across multiple specialties. The small profile of the system also provides space for surgical assistants and nurses to access the patient and provide assistance during robotic operation. Robotic arms were equipped with several strategically placed user interfaces making docking and manipulation during the procedure easy.

To allow for an expedited switch between standard laparoscopy and robotic assistance, the surgeon needs to work in a sterile gown on a sterile console to approach and leave the operating field any time during the procedure. This expedited switch contrasts with conventional fully robotic platforms where the surgeon needs to scrub and the robot to be undocked before conversion is possible. As opposed to conversion in this traditional robotic setting, Dexter allows to switch within seconds. Dexter enhanced minimally invasive surgery by offering the possibility to the surgeon to remain sterile when commanding the robot from the surgeon console and ensuring a quick access to the patient bed as needed. Each of the robot arms may be folded on command without compromising their docking. This on-demand approach of robotic-assisted surgery is particularly advantageous during colectomies. CVL requires precise dissection and movements. The articulated instruments and the increased precision simplify dissection. In addition, the full robotic endoscope control from the console improves image stability and quality including surgeon ergonomics. However, for medialization of the colon a robotic platform is not needed. This may be performed faster by switching to standard laparoscopy. The robotic arms fold back providing a sufficient workspace for the surgeons for standard laparoscopy. We applied this concept of robotics for CVL and suturing of the defect after stapled anastomosis and standard laparoscopy for mobilization of the colon in the herein described patients. The range of movements of the robotic arms (see Fig. 4) allow in theory also for robotic mobilization of the colon. However, we believe that this part of the operation can be performed faster and more efficiently by standard laparoscopy as it does not require the precision of a robotic device. Larger studies with more patients will define in the future for each operation the steps performed by Dexter or by standard laparoscopy. As switching from laparoscopy to robotics and back can be done within seconds the surgeon can choose at any time which parts of the operation he wants to do with Dexter or not. This flexibility is a real advantage in our opinion.

Furthermore, the distribution of the working trocars is key to allow for comfortable laparoscopic navigation in the third space. While the trocar setup of conventional fully robotic platforms is unfavorable and not suited for a laparoscopic trocar setup, the trocar positions in the Dexter system is close to our standard laparoscopic setup.

The endoscope arm of the Dexter system allows the surgeon to fully control camera navigation from the console. However, camera control can be transferred to manual any time, to easily carry on with the procedure bedside. While this offers the advantage of improved visualization due to full endoscope control, the system remains flexible on-demand.

Centers can use their own RF generator, insufflator and also their own 3D endoscopic system. The camera arm can cope with any available system. Furthermore, standard tools such as staplers and clip appliers can be used from any institutional inventory. The platform offers a full set of robotic instruments. These instruments are single use. Only the incision pointer used for docking, the handles of the surgeon console and the tool only used in case of manual instrument release need sterilization using standard processes. This is another major advantage for institutions. We highly recommend using a 3D endoscopic system to allow for better depth perception and safer orientation in the abdomen.

Currently, Dexter is only available in Europe (CE marked) and is used for gynecological procedures, prostatectomies [7] and partial nephrectomies, hernia surgery and colorectal resections and ventral mesh rectopexies.

In summary, the on-demand robotics concept allows to switch between the robotic and laparoscopic platform at the surgeons’ discretion within seconds. Thus, combining the best of both approaches. The system is compatible with standard OR equipment, which helps to limit expenses related to its implementation. After this preliminary experience, our group is leading a prospective feasibility and safety trial including the different surgical specialties in general surgery, urology and gynecology.

## References

[1] Abd El Aziz MA, Grass F, Behm KT, Shawki S, D'Angelo AL, Mathis KL (2021). Trends of complications and innovative techniques' utilization for colectomies in the United States. Updates Surg.

[2] Sheetz KH, Norton EC, Dimick JB, Regenbogen SE (2020). Perioperative outcomes and trends in the use of robotic colectomy for medicare beneficiaries from 2010 through 2016. JAMA Surg.

[3] Hinrichs-Krapels S, Ditewig B, Boulding H, Chalkidou A, Erskine J, Shokraneh F (2022). Purchasing high-cost medical devices and equipment in hospitals: a systematic review. BMJ Open.

[4] Jayne D, Pigazzi A, Marshall H, Croft J, Corrigan N, Copeland J (2017). Effect of robotic-assisted vs conventional laparoscopic surgery on risk of conversion to open laparotomy among patients undergoing resection for rectal cancer: the ROLARR randomized clinical trial. JAMA.

[5] Crippa J, Grass F, Dozois EJ, Mathis KL, Merchea A, Colibaseanu DT (2020). Robotic surgery for rectal cancer provides advantageous outcomes over laparoscopic approach: results from a large retrospective cohort. Ann Surg.

[6] Feng Q, Yuan W, Li T, Tang B, Jia B, Zhou Y (2022). Robotic versus laparoscopic surgery for middle and low rectal cancer (REAL): short-term outcomes of a multicentre randomised controlled trial. Lancet Gastroenterol Hepatol.

[7] Bohlen D, Gerber R (2023). First ever radical prostatectomy performed with the new dexter robotic system. Eur Urol.

[8] Thillou D, Robin H, Ricolleau C, Benali NA, Forgues A, Emeriau D (2023). Robot-assisted radical prostatectomy with the dexter robotic system: initial experience and insights into on-demand robotics. Eur Urol.

[9] Hubner M, Larson DW, Wolff BG (2012). “How I do it”–radical right colectomy with side-to-side stapled ileo-colonic anastomosis. J Gastrointest Surg.

[10] Grass F, Slieker J, Jurt J, Kummer A, Sola J, Hahnloser D (2017). Postoperative ileus in an enhanced recovery pathway-a retrospective cohort study. Int J Colorectal Dis.

